# Gene Expression Analyses of the Spatio-Temporal Relationships of Human Medulloblastoma Subgroups during Early Human Neurogenesis

**DOI:** 10.1371/journal.pone.0112909

**Published:** 2014-11-20

**Authors:** Cornelia M. Hooper, Susan M. Hawes, Ursula R. Kees, Nicholas G. Gottardo, Peter B. Dallas

**Affiliations:** 1 Brain Tumour Research Program, Telethon Kids Institute, University of Western Australia, Subiaco, Western Australia, Australia; 2 Centre of Excellence in Computational Systems Biology, ARC Centre of Excellence in Plant Energy Biology, University of Western Australia, Perth, Western Australia, Australia; 3 Monash Institute of Medical Research, Monash University, Clayton, Victoria, Australia; 4 Division of Children's Leukaemia and Cancer Research, Telethon Kids Institute, University of Western Australia, Subiaco, Western Australia, Australia; 5 Department of Paediatric Oncology and Haematology, Princess Margaret Hospital for Children, Subiaco, Western Australia, Australia; Universitat Pompeu Fabra, Spain

## Abstract

Medulloblastoma is the most common form of malignant paediatric brain tumour and is the leading cause of childhood cancer related mortality. The four molecular subgroups of medulloblastoma that have been identified – WNT, SHH, Group 3 and Group 4 - have molecular and topographical characteristics suggestive of different cells of origin. Definitive identification of the cell(s) of origin of the medulloblastoma subgroups, particularly the poorer prognosis Group 3 and Group 4 medulloblastoma, is critical to understand the pathogenesis of the disease, and ultimately for the development of more effective treatment options. To address this issue, the gene expression profiles of normal human neural tissues and cell types representing a broad neuro-developmental continuum, were compared to those of two independent cohorts of primary human medulloblastoma specimens. Clustering, co-expression network, and gene expression analyses revealed that WNT and SHH medulloblastoma may be derived from distinct neural stem cell populations during early embryonic development, while the transcriptional profiles of Group 3 and Group 4 medulloblastoma resemble cerebellar granule neuron precursors at weeks 10–15 and 20–30 of embryogenesis, respectively. Our data indicate that Group 3 medulloblastoma may arise through abnormal neuronal differentiation, whereas deregulation of synaptic pruning-associated apoptosis may be driving Group 4 tumorigenesis. Overall, these data provide significant new insight into the spatio-temporal relationships and molecular pathogenesis of the human medulloblastoma subgroups, and provide an important framework for the development of more refined model systems, and ultimately improved therapeutic strategies.

## Introduction

Medulloblastoma is the most common type of malignant brain tumor affecting children. Although the prognosis for patients has improved markedly in the last thirty years, the outlook for those with metastatic or recurrent disease remains poor [1], and for many patients post-treatment sequelae often result in significant long-term intellectual and/or developmental impairment and associated psychosocial problems [2]. These issues highlight the pressing need for more effective treatment strategies with less damaging collateral effects. Significant progress has been made towards these goals in the last five years, with an improved understanding of the molecular profiles underpinning the histological and clinical heterogeneity of medulloblastoma. The current consensus points to the existence of at least four molecular subgroups [3], [4] including a predominantly desmoplastic Sonic Hedgehog (SHH) subgroup, a Wingless (WNT) classic subgroup, and two other less clearly defined subgroups, Group 3 and Group 4, which exhibit classic and large-cell/anaplastic histology and gene signatures characterized by activated photoreceptor or neuronal receptor signaling, respectively. The accruing data reveal that medulloblastoma subgroups arise in distinct regions of the posterior fossa [5]–[7] are associated with characteristic genetic aberrations [8], and may be derived from distinct cells of origin.

Substantial evidence has been generated in the last decade to support the hypothesis that for at least some cancers, a minority population of cells with stem cell like properties may be primarily responsible for driving tumor growth [9]. Indeed, previous reports revealed that human medulloblastoma contain a small proportion of cells expressing the neural stem cell (NSC) markers CD133^+^ and Nestin^+^ (NES), which were the only tumor cell population displaying the hallmark stem cell characteristics of self-renewal and differentiation [10]–[12]. Critically, only the CD133^+^ cells were capable of regenerating human medulloblastoma that were phenotypically identical to the original tumor in xenograft assays [10]. Several murine models have demonstrated that medulloblastoma with characteristics similar to human subgroups can be generated through the genetic manipulation of NSCs. Importantly, these studies highlight the critical relationship between the differing molecular pathogenesis of the various medulloblastoma subgroups and the spatio-temporal origins of the NSCs in which the initiating tumorigenic events occur. For example, expression of mutant n-Myc in E16 cerebellar NSCs induces SHH medulloblastoma, while expression of the same mutant at P0, produces medulloblastoma with human Group 3 characteristics [13]. SHH and Group 3 medulloblastoma have also been generated using other approaches, including deletion of *Ptch1* [14] in GFAP+ cerebellar NSCs, and combined inactivation of Rb and p53 in CD133+ NSCs [15], respectively. Finally, medulloblastoma that mimic the human WNT subgroup were initiated by activating *CTNNB1* mutations in dorsal brainstem precursors [5]; however, GFAP+ cerebellar stem cells may also be the cell of origin for some WNT group tumors [16]. In murine tumor models, transcriptional profiling comparisons suggest that WNT and SHH-medulloblastoma arise during early embryonic development equivalent to the first 6 weeks of human embryogenesis [17]. Similar human studies are lacking due to the ethical issues associated with obtaining human embryonic and foetal brain tissues, and little is known of the spatio-temporal origins of human Group 3 and Group 4 medulloblastoma. For SHH and WNT medulloblastoma, gene expression profiles overlap with stem cell regulatory pathways [18]–[20] while activated expression of later neuronal signaling and maintenance pathway genes, is characteristic of Group 3 and Group 4 medulloblastoma [21].

To define the spatio-temporal relationships of human medulloblastoma subgroups from a neuro-developmental perspective, we aligned the transcriptional profiles of two primary medulloblastoma cohorts with the profiles of early human neural tissues and cell types that represent different stages of embryonic and early foetal human neural development. Our data demonstrate the molecular relationships between medulloblastoma subgroups and normal neural cell types at distinct developmental stages, and highlight subgroup specific deviation of expression profiles away from normal neuronal lineage differentiation patterns, which may contribute to the pathogenesis of medulloblastoma subgroups.

## Materials and Methods

### Patient specimens

The study was approved by the Princess Margaret Hospital for Children (PMH) Human Ethics Committee (Perth, Western Australia) and written consent was obtained for the use of all samples for research. The Australian medulloblastoma cohort (AU-MB) included 19 childhood medulloblastoma specimens obtained from PMH (n = 14) and the Westmead Tumour Bank (WTB), Sydney, New South Wales (n = 5). For more details about the AU-MB cohort and developmental control samples refer to Table S1. Normal fetal germinal matrix (NFGM) samples were obtained from the lateral sub-ventricular zones of two 16 week old aborted male fetuses at King Edward Memorial Hospital, Perth, Western Australia. Human normal fetal brain (NFB) consisted of total RNA pooled from 50 individuals (embryonic week 22–33) and was purchased from BD Bioscience (Palo Alto, CA, USA). Expression profiles of 46 independent medulloblastoma samples [22] were obtained from St. Jude Children's Research Hospital (SJ-MB, http://StJuderesearch.org).

### Neural stem cell culture

Human neurospheres were derived from embryonic stem cell (hESC) lines hES3, hES4 (ES Cell International, Singapore, http://www.escellinternational.com) and MEL1 (StemCore, Melbourne, VIC, Australia, http://www.stemcore.com.au), derived from three different individuals, using a protocol described previously [23], [24]. In brief, hESCs were co-cultured for 14 days on a layer of mitomycin-C-treated mouse embryo fibroblasts in the presence of the *BMP2*-antagonist, Noggin (500 ng/ml; R&D Systems – Bioscientific, Gymes Australia). Cell clumps were mechanically excised from Noggin-treated colonies and transferred to 96-well ultra-low adherence plates (Corning, Corning, NY, USA). Neurospheres were cultured up to 12 weeks in neural basal medium (NBM) (Gibco, Invitrogen, Carlsbad, CA, USA) supplemented with N2 (Gibco), B27 (Gibco), Penicillin/Streptomycin (Gibco), L-Glutamine (Sigma, St Louis, MO, USA), Interferrin-Transferrin-Selenium (Gibco), EGF (20 ng/ml; Sigma) and bFGF (20 ng/ml; Chemicon, Temecula, CA, USA). Growth factors were added fresh every 2–3 days during media change. Neurospheres were quartered and replated for propagation as described previously [25].

### Isolation of CD133+ NSCs

Neurospheres consisting of ∼30% CD133+ NSCs and ∼70% CD133- neural progenitor cells (NPCs) [23], [26], were dissociated using a trypsin-based neural tissue dissociation kit (Miltenyi Biotec, Bergisch Gladbach, Germany). Cells were centrifuged (400×g) and resuspended in FACS buffer (PBS supplemented with 0.5% bovine serum albumin (BSA) (Sigma) and 2 mM ethylene diamine triacetic acid (EDTA) (Sigma). Cells were blocked with fragment crystallisable region (FcR) (Miltenyi Biotec) for three min at room temperature and incubated with AC133/1-Phycoerythrin (PE) and CD133/2(C293)-PE antibodies (Miltenyi Biotec) on ice for 20 min. As negative controls, cells were incubated with IgG_2b_-PE (Santa Cruz Biotechnology, Santa Cruz, CA, USA) at the same concentration, or antibodies were omitted. Excess antibody was removed by washing in FACS buffer, and samples were isolated using an ARIA-I flow cytometer (BD Biosciences, San Jose, CA, USA). CD133^+^ NSCs were isolated from neurosphere cell lines MEL1, hES3, and hES4, at 90% or greater purity (Table S1). The purity of CD133^-^ NPCs derived from the same cell lines was consistently greater than 99%. The expression of *PROM1* (p<0.05) and *NES* (p<0.01) was significantly higher in the CD133+ compared to the CD133-depleted samples consistent with successful enrichment of CD133^+^ NSCs (data not shown).

### Tissue processing and mRNA extraction

Tumour and foetal brain tissue were disrupted using an immersion processor and cells were washed in PBS and pelleted before resuspension in TRIzol (Invitrogen, Carlsbad, CA, USA). After 5min of incubation at RT, 0.2 ml of chloroform (AnalaR, Merck, Whitehouse Station, NJ, USA) was added to each 1 ml TRIzol. The phases were mixed and centrifuged (13000 rpm) for 15 min at 4°C. The upper phase was collected and adjusted to 35% ethanol by adding 100% pure ethanol (AnalaR, Merck, Whitehouse Station, NJ, USA). The solution was applied to an RNeasy column (Qiagen, Germantown, MD, USA) and RNA was purified according to the manufacturer's instructions. The RNA was eluted using 50 µl diethyl pyrocarbonate (DEPC)-treated water and precipitated by the addition of 0.1 volume NaOAc (Sigma, St Louis, MO, USA), 2.5 volume of 100% ethanol and 1 µl glycogen (Ambion, Foster City, CA, USA). After 1 hr at −20°C, the samples were centrifuged (13000 rpm) for 20 min at 4°C. The RNA pellet was washed twice with 80% ethanol and air-dried prior to resuspension in 10 µl DEPC-treated water. RNA integrity was measured with an Agilent 2100 Bioanalyser (Agilent, Santa Clara, CA, USA).

### Gene expression analysis

The gene expression profiles of the AU-MB cohort were generated using human U133A arrays (Affymetrix, Santa Clara, CA, USA) and a protocol described previously [27]. Annotated raw and processed mRNA expression data are available NCBI GEO accession no. GSE62600.

The SJ-MB gene expression profiles are publically available and were generated using the Affymetrix human U133A_v2 array [22]. Statistical analysis was performed in R 2.14.0 (http://www.r-project.org) and bioconductor version 2.10 (http://www.bioconductor.org). Affymetrix cel files were normalized using robust multi-array average (Package: *affy*). For statistical analysis, *simpleaffy, annotate, hgu133acdf, limma, vegan* and *matrixStats* packages were used. The Student's T-test was performed pair-wise between all sample groups with p<0.05 deemed as significant. For comparison to NFB (one value only) the NFB expression value was tested for inclusion or exclusion from medulloblastoma sample distribution using standard deviation (stdev) as membership cut off. Tables with mean ± stdev, median, variance, fold change, Student's T-test values and σ-fold deviation were generated for all medulloblastoma subgroups *versus* all control samples. Principal component analysis (PCA) and global hierarchical clustering was performed in R (Package: *stats*). The AU-MB cohort was classified into molecular subgroups using principal components analysis (PCA) and alignment to SJ-MB subgroups as previously determined [28]. For classification, we used 20 of 24-classifier genes (*LEF1, RUNX2, DCX, MAB21L1, PTCH1, PDLIM3, NEUROG1, DLL3, PDGFA, FOXG1B, GRM1, VAMP4, CDKN1C, SERPINF1, NRL, CRX, NMNAT2, SMARCD3, GABRA5* and *DCC*) reported by Kool et al (2008) [28]. The remaining four genes, *OTX2, LEMD1, PTPN5* and *ZNF179,* were not represented on the U133A array used in this study. The 24-classifier genes were identified from the analysis of Affymetrix gene expression data generated from 62 medulloblastoma that were further validated in an independent cohort of 46 specimens. The 24-classifier gene set represented the smallest set of genes with the greatest variance in expression, which also showed the most robust cluster result with the highest support from bootstrap analysis [28]. For the various sample groups, cluster coordinate centroids were calculated and centroid distance was generated as geometric vector length. Results were graphed using R, Excel version 14.0.0 (Microsoft) and Adobe Illustrator (Creative Suite 5.5, Adobe).

### Developmental intersect analysis (DIA)

The medulloblastoma expression intersect database was constructed using SequelPro 0.9.9.1 (http://www.sequelpro.com), and tabular data were imported from R. Probe sets ranging below 200 intensity units were deemed below detection limit. Using structured query language (SQL), the compiled Student's T-test result database was queried for significantly differentially expressed transcripts in the medulloblastoma subgroups in comparison to all controls using a step-wise intersect protocol. Query results were analyzed using Ingenuity Pathway Analysis (IPA, http://www.ingenuity.com).

### Weighted Gene Co-expression Network Analysis (WGCNA)

Genes operating in neighbourhoods (clusters) are often co-expressed [29], and genes clusters can form associated cluster families. Mathematically, the association strength acts as a distance measure for clusters in networks, which organizes clusters spatially within the network. This can be graphed as dendograms representing clusters as branches. When relating non-expression characteristics (traits) to such networks, their association will reveal relationships with specific functional neighbourhoods. Co-expression network clustering was performed in R (Package: *WGCNA*) according to the protocol of Langfelder and Horvath [30]. In brief, medulloblastoma gene expression data derived from both cohorts were filtered and low signal probe sets with low expression variation were omitted. Probe sets with the extension *x_at* were omitted. The remaining 6052 probe sets were used for approximation to the scale-free small world network model. Genes were grouped into clusters of significantly co-expressed gene sets using Pearson statistics. The correlation of clusters was used to construct cluster families within the network. For developmental alignment, co-expression network clusters were correlated to mean intensity of developmental control samples in order of their alignment derived from PCA and compared to alternative order possibilities. Pearson correlation coefficients and p values were generated for each alignment order and cluster. Cluster families were constructed using cluster Eigengenes (the normalized linear combination of genes with largest variance in a population), considered as the representative gene expression pattern of each cluster. For association of medulloblastoma subgroups and cluster families, molecular subgroup membership of medulloblastoma samples was correlated to clusters using a binary matrix. Genes within co-expression clusters of interest were analyzed using SQL and IPA for over-expression and pathway enrichment as described above.

## Results

### Medulloblastoma molecular subgroups align along a neuronal differentiation continuum

**Figure 1:**
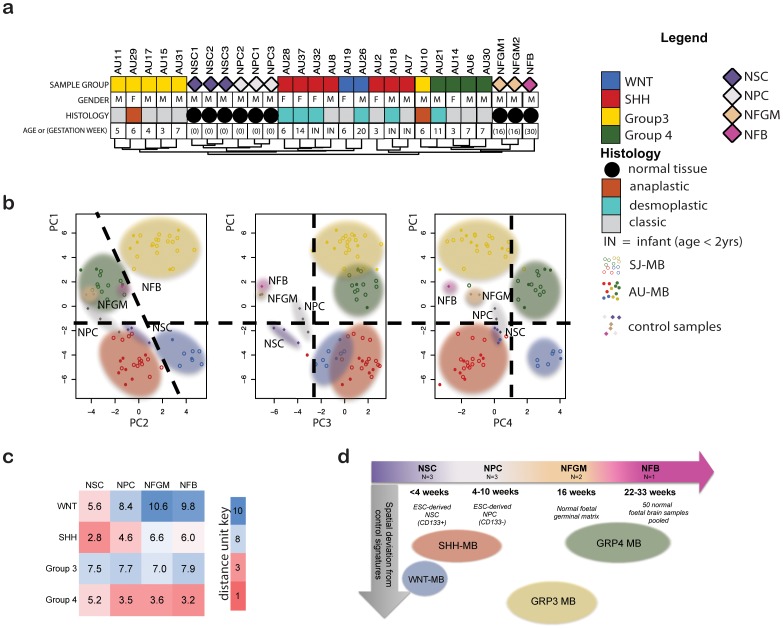
Alignment of molecular subgroups to developmental controls. **a.** Unsupervised clustering using global expression signatures of the AU-MB cohort and developmental controls is displayed with patient gender, age and tumor histology features. **b.** Multivariate analysis of combined AU-MB and SJ-MB cohorts using 20-gene molecular subgroup classifiers. Spatial distribution of medulloblastoma subgroups and developmental control samples is graphed for the first four largest variances. Medulloblastoma subgroup variance in the context of control samples is best displayed by PC1, 2 and 4. **c.** Distance measures of control *versus* medulloblastoma subgroups by geometric distance of group centroid in the variance dimensions PC1 and PC2. The arbitrary distance units were graphed on a blue (far) to red (close) scale. **d.** Interpretation of spatial and temporal association of clustering alignment analyses. Geometric distance of medulloblastoma subgroups to all control samples groups is schematically graphed in distance along the temporal brain development axis (x-axis) and spatial axis (y-axis, average distance of subgroup to all control groups). *AU-MB – Australian medulloblastoma cohort (solid symbols), NFB - normal fetal brain, NFGM - normal fetal germinal matrix, NPC - neural progenitor cells, NSC - neural stem cells, PC – principal component, SJ-MB – St Jude medulloblastoma cohort (open symbols)*.

The current consensus includes at least four subgroups of medulloblastoma with distinct molecular and clinical features [3]. Consistent with this, hierarchical unsupervised clustering confirmed the presence of at least four groups with clusters containing WNT and SHH medulloblastoma in the AU-MB cohort (Fig. 1a). Similarly, multivariate analysis of the combined AU-MB and SJ-MB cohorts based on either the 65-gene classifier [21] (data not shown) or 20-gene classifier [28] for molecular subgroups, resulted in the AU-MB clustering with the four molecular subgroups reported previously [22] in the SJ-MB cohort (Fig. 1b). Based on these data, we combined all AU-MB and SJ-MB specimens to generate molecular cohorts of WNT (n = 8), SHH (n = 21), Group 3 (n = 22) and Group 4 (n = 14) medulloblastoma for further subgroup-specific analyses. PCA revealed that the first five variance components described greater than 80% of the variance in the data set (Fig. S1), with only the first four being of discriminative value. The first component captured most of the variance describing the difference between WNT and SHH subgroups that clustered close to NSCs, and Group 3 and 4 clustering closer to NFGM and NFB (Fig. 1b). In contrast, the second and fourth PCs described distinct differences between WNT and SHH medulloblastoma as well as those between Group 3 and Group 4. The third PC mostly described the differences between any control and any medulloblastoma sample and was not used for assessing the spatial relationships between medulloblastoma subgroups with respect to controls. However, the variation accounted for by the third PC reflected differences between neoplastic and non-neoplastic samples, and intersect analyses revealed that this variance was derived from differential expression of chromatin regulators (data not shown). The spatial distance between sample groups was established using the geometric distance of group centroid for PC1, 2 and 4 (Fig. 1c). This revealed that WNT and SHH subgroups clustered closest to NSCs and NPCs, indicating less variance between these medulloblastoma groups and embryonic cell types. Group 4 clustered closest to NFB, then NFGM, highlighting a closer relationship to an early fetal cell type. Both hierarchical clustering and multivariate analysis showed that Group 3 medulloblastoma did not cluster particularly close to any early developmental control, with the nearest neighbor being NFGM by PCA. We brought together the PC data (Fig.1b), additional statistical parameters (cluster scatter and cluster shape), as well as known biology to summarise the spatial relationships between controls and medulloblastoma subgroups, and project them schematically onto a brain development timeline (Fig. 1d). This represents a spatio-temporal snapshot of the medulloblastoma developmental signatures aligned to control samples in this study.

The alignment of NSCs, NPCs, NFGM and NFB with WNT, SHH, Group 3 and Group 4 medulloblastoma was tested using gene signatures organized into functional genetic neighborhoods. A gene co-expression network was constructed using all medulloblastoma samples generating 27 gene clusters that formed at least five cluster families (A–E) (Fig. S2). To test the proposed medulloblastoma alignment to controls as shown schematically (Fig. 1d), possible alignments were treated as traits and associated with the medulloblastoma network. Cluster family E was the genetic neighborhood most descriptive for alignment analysis and was strongly correlated with the association of WNT/SHH subgroups with embryonic samples and Groups 3 and 4 with foetal samples. Cluster correlation increased when WNT medulloblastoma were associated with NSCs instead of NPCs, and Group 3 with NFGM instead of NFB. Consistent with PCA, transcripts from the functional neighborhood E correlated most strongly (Pearson coefficient  = 0.87, p<2.2×10^−16^). Since this neighborhood contains neuronal differentiation gene sets, medulloblastoma subgroups (WNT <SHH < Group 3< Group 4) may represent stages of a neuronal differentiation continuum.

### WNT and SHH pathway activation is higher in WNT and SHH medulloblastoma than NSCs

**Figure 2:**
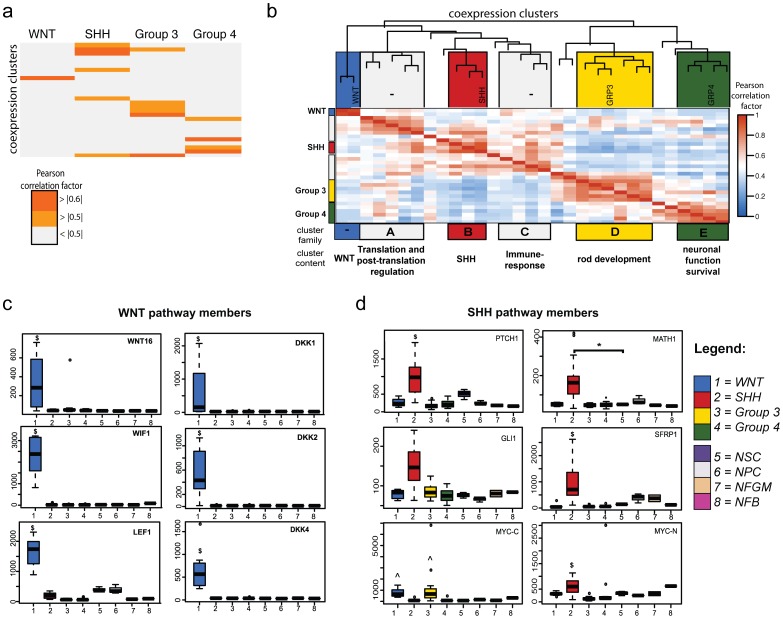
Gene co-expression and module association with medulloblastoma molecular subgroups. **a.** The gene co-expression network was constructed for the combined medulloblastoma cohorts. Gene expression from each co-expression cluster was correlated to medulloblastoma subgroup. Correlation strength (Pearson correlation coefficient) across co-expression clusters and medulloblastoma subgroups was graphed using a grey to orange color scale. **b.** Using the association between clusters as a distance measure, cluster family topology was generated and the co-expression network was graphed as a dendrogram describing gene cluster relatedness. The main enriched pathways in cluster families are shown below and indicate medulloblastoma subgroup-associated functional gene neighborhoods. Expression of major contributors to cluster association for **c.** WNT and **d.** SHH medulloblastoma are shown in Box-Whisker plots for each sample group included in this study. * −p<0.05, $ −p<0.05 compared to all control groups with n>1 and exclusive for molecular subgroup, ∧ −p<0.05 compared to all control groups but not exclusive for medulloblastoma subgroup, NFB - normal fetal brain, NFGM - normal fetal germinal matrix, NPC - neural progenitor cells, NSC - neural stem cells.

To identify functional neighborhoods descriptive for medulloblastoma subgroups, the membership of each medulloblastoma in a molecular subgroup was used as a trait. Analysis of associations between co-expression network clusters and membership (traits) showed that distinct subgroups are significantly correlated with distinct gene clusters of the co-expression network (Fig. 2a). Taking the network organization of clusters into account, medulloblastoma subgroups associated with cluster families (Fig. 2b) that contained distinct genetic functional neighborhoods. The associated gene sets of the clusters represent major functional genetic connections underlying medulloblastoma expression profiles and provide clues to mechanisms of pathogenesis.

WNT medulloblastoma were most strongly associated (Pearson coefficient  = 0.92; p<7e^−27^) with only one cluster of co-expressed genes enriched for WNT/β-catenin signaling (p<0.002), and WNT-related cancer gene sets (BCC, GBM, ovarian cancer, p<0.01) (Table. 1) and not any other cluster or cluster family. Transcripts contributing significantly to these enrichments encoded *WNT16, DKK1* and *LEF1* that are specifically over-expressed in WNT medulloblastoma irrespective of developmental control comparison (Fig. 2c). These gene sets overlapped strongly with intersect analyses selecting for exclusively over-expressed transcripts in WNT medulloblastoma irrespective of control comparison (Table S3, S4). In contrast, SHH medulloblastoma associated with cluster family B, containing genes enriched for SHH signaling (p = 0.01), as well as extracellular matrix-affiliated pathways (Table. 1). The main contributors to these enrichments are *GLI2, PTCH1, PTCH2, AXIN, MATH1* and a number of collagen genes. Some of these genes are exclusively over-expressed in SHH medulloblastoma (Fig. 2d). These data demonstrate that the co-expression network alignment and intersect analysis strategy was able to filter for known deregulation of WNT and SHH pathway members in WNT and SHH medulloblastoma, respectively. These two gene sets represent distinct stem cell maintenance pathways that are strongly linked and exclusively over-expressed in these subgroups. Of note, the expression of most WNT and SHH pathway members was significantly higher in WNT or SHH-medulloblastoma than in CD133^+^ NSCs (Fig. 2c, d). In contrast, members of the NOTCH signaling pathway, another major stem cell maintenance pathway, were not highly expressed in any medulloblastoma subgroup compared to NSCs and their immediate derivatives (Fig. S3).

**Table 1:** Pathway enrichment of medulloblastoma-associated co-expression cluster families.

		Enriched pathways	p<	*Contributing genes*
**WNT brown cluster**	**(342 probes)**	Wnt/β-catenin Signaling	0.002	*FZD10, CDH2, WIF1, DKK4, WNT16, DKK2, LEF1, DKK1, FZD7*
		Glioblastoma Multiforme Signaling	0.003	*FZD10, PDGFRA, CDK6, WNT16, PLCB1, LEF1, PDGFC, FZD7*
		Basal Cell Carcinoma Signaling	0.006	*FZD10, BMP4, WNT16, LEF1, FZD7*
		Ovarian Cancer Signaling	0.006	*FZD10, LHCGR, WNT16, LEF1, MMP2, PDGFC, FZD7*
**SHH cluster family B)**	**(358 probes)**	Hepatic Fibrosis	0.002	*COL1A2, COL1A1, IGFBP4, FGFR1, IGFBP5, PGF, COL3A1, EGFR*
		Molecular Mechanisms of Cancer	0.003	*RAP2B, A XIN1, ARHGEF7, PTCH1, PTCH2, FOXO1, GAB1, RHOB, MAPK10, BID, MAP2K3, CDK2, CTNND1*
		Basal Cell Carcinoma Signaling	0.005	*GLI2, AXIN1, PTCH1, DVL3, PTCH2*
		Sonic Hedgehog Signaling	0.010	*GLI2, PTCH1, PTCH2*
**Group 3 cluster family D**	**(508 probes)**	Phototransduction Pathway	3.89E−10	*PDE6G, GUCA1A, GNGT1, GNB5, PRKAR2A, PDE6H, GNB3, GNAT1, RHO, PDC, SAG, CNGB1, RCVRN*
		Protein Kinase A Signaling	0.0087	*RAF1, PDE6G, MYL6, PTP4A3, GNB5, PRKAR2A, MYL6B, PTPRF, PDE6H, GNB3, RHO, PPP1R10, TGFB1, TGFB3, CNGB1, TNNI1, CDC25A, MTMR3*
		Cyclins and Cell Cycle Regulation	0.0138	*RAF1, TGFB1, ABL1, TGFB3, HDAC5, CDC25A*
		Estrogen Receptor Signaling	0.0141	*CTBP1, RAF1, POLR2A, NCOR2, MED24, TRRAP, MED27, SMARCA4*
**Group 4 cluster family E**	**(343 probes)**	Semaphorin Signaling in Neurons	1.23E−05	RND2, CRMP1, DPYSL3, DPYSL4, DIRAS3, RAC1, FNBP1
		Glioblastoma Multiforme Signaling	7.41E−05	RND2, SRC, TSC1, DIRAS3, IGF1R, RAC1, PLCL2, CDKN1B, PLCH2, FNBP1
		Axonal Guidance Signaling	1.20E−04	NTF3, ITSN1, EPHB2, TUBB2A, RAC1, PLXNA2, PLCL2, GNAZ, PLCH2, NCK2, SRGAP3, PRKAR2B, TUBA1A, PTPN11, PPP3CB, GNAO1, BAIAP2
		Gap Junction Signaling	1.23E−04	DBN1, HTR2C, SRC, PRKAR2B, TUBA1A, PPP3CB, ADCY1, TUBB2A, PLCL2, PLCH2
		Actin Nucleation by ARP-WASP Complex	1.91E−04	RND2, NCK2, DIRAS3, BAIAP2, RAC1, FNBP1
		Cardiac Hypertrophy Signaling	0.001	RND2, PRKAR2B, PPP3CB, DIRAS3, GNAO1, ADCY1, IGF1R, PLCL2, GNAZ, PLCH2, FNBP1
		Ephrin B Signaling	0.001	NCK2, ITSN1, EPHB2, GNAO1, RAC1, GNAZ
		RhoGDI Signaling	0.001	RND2, SRC, DIRAS3, GNAO1, RAC1, PIP4K2B, CDH18, GNAZ, FNBP1
		Sphingosine-1-phosphate Signaling	0.001	RND2, DIRAS3, ADCY1, RAC1, PLCL2, PLCH2, FNBP1
		Regulation of Actin-based Motility by Rho	0.001	RND2, DIRAS3, BAIAP2, RAC1, PIP4K2B, FNBP1
		Clathrin-mediated Endocytosis Signaling	0.002	SRC, ARRB1, PPP3CB, EPHB2, SH3GL3, RAC1, HIP1, FGF13, HIP1R
		Neuregulin Signaling	0.002	SRC, PTPN11, ERBB4, CDKN1B, STAT5B, CDK5R1

Group 3 photoreceptor gene neighbourhood signatures are reminiscent of rod, but not cone, precursor cells at weeks 10 to 15 of human neurodevelopment

**Figure 3:**
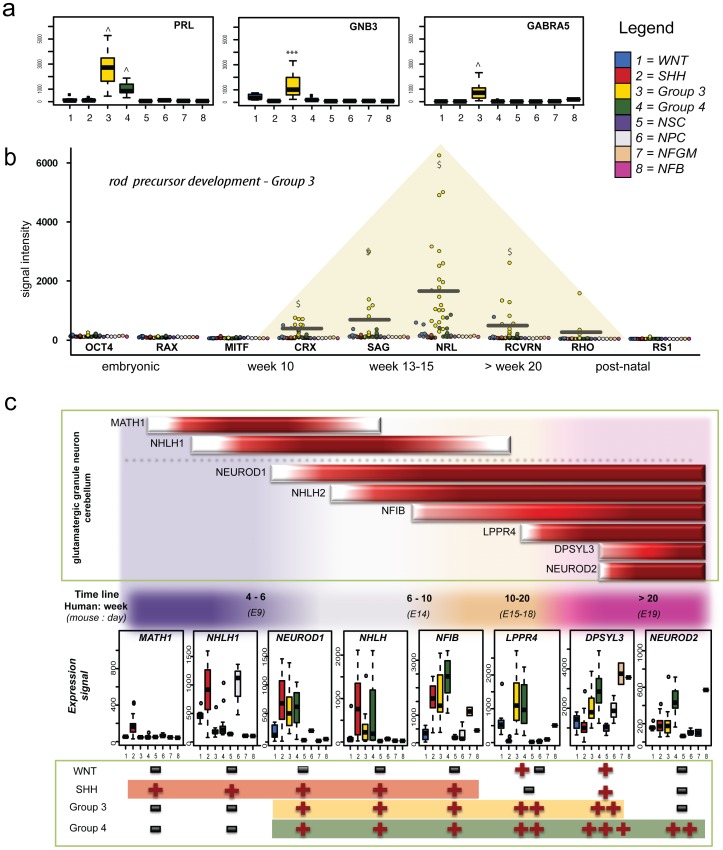
Developmental alignment of Group 3 and Group 4 medulloblastoma. **a.** The expression levels of selected exclusively Group 3 over-expressed photoreceptor and glutamate signaling genes were graphed for all sample groups using Box-Whisker plots. **b.** Expression of rod photoreceptor genes transiently and terminally expressed during retinal differentiation was graphed for all medulloblastoma and control samples. The mean expression and the time window of active rod gene expression for Group 3 medulloblastoma is indicated and emphasized by yellow shaded overlay. **c.** Developmental alignment of key transcription factors expressed during glutamatergic granule neuron development. Expression time frame during normal cerebellar maturation is indicated schematically in the top panel. Gene expression for medulloblastoma and control groups is shown in box-whisker plots. Developmental expression windows of time stamp genes in medulloblastoma subgroups are schematically displayed in the bottom panel. *** −p<0.001, $ –p<0.05 compared to all control groups with n>1 and exclusive for molecular subgroup, ∧ −p<0.05 compared to all control groups but not exclusive for medulloblastoma subgroup, NFB - normal fetal brain, NFGM - normal fetal germinal matrix, NPC - neural progenitor cells, NSC - neural stem cells.

Using the above analysis strategy, we were able to identify genes and pathways that were differentially regulated in Group 3 medulloblastoma irrespective of the control group comparison (Table S2, S3). Pathways identified from these analyses included the photo transduction pathway (p = 2.51×10^−12^), protein kinase A signaling (p = 0.002), glutamate (**p<0.01) and glucocorticoid (*p<0.02) receptor signaling 3 (Table. 1). Some individual members of these pathways, including *GNB3, GNB5, GABRA5, RCVRN* and *SAG* were exclusively over-expressed in Group 3 medulloblastoma (Fig. 3a). In comparison to previous studies reporting general photoreceptor pathway activation as a hallmark of Group 3 medulloblastoma, this study revealed that Group 3 expression profiles were characteristic of early rod cell differentiation, whereas cone lineage genes were not expressed (Fig. S4). Rod development genes expressed by Group 3 medulloblastoma were representative of week 10–20, corresponding to the mid fetal phase of retinal or pineal development. The early rod precursor-specific transcripts, *CRX* and *NRL* were exclusively and strongly over-expressed by Group 3 medulloblastoma (Fig. 3b). Further, rhodopsin (*RHO*) and retinoschisin *1* (*RS1*) normally highly expressed by functional rod cells post-natally, were weakly expressed, but detectable in most Group 3 samples. Temporally, this stage of early rod determination is best represented by NFGM in this study. Despite the large spatial deviation between Group 3 medulloblastoma and any control group in previous cluster analyses, NFGM remained the most suitable control for comparison within this data set. A selection of the most differentially expressed genes in Group 3 compared to NFGM (including *NEUROD1, MYC, SOX2*, and *DLX*) is listed in Table 2.

**Table 2:** Over-expressed transcripts in Group 3 and Group 4 medulloblastoma.

	*Over-expressed candidate genes*			*Tumor suppressor candidate genes*		
	Probe ID	gene	fch	*Probe ID*	*description*	*fch*
Group 3 *versus* NFGM	206163_at	mab-21-like 1 (MAB21L1)	76.7	*207147_at*	*distal-less homeobox 2 (DLX2)*	*−62.0*
	205445_at	prolactin (PRL)	45.1	*209988_s_at*	*achaete-scute complex homolog 1 (ASCL1)*	*−32.0*
	202310_s_at	collagen, type I, alpha 1 (COL1A1)	43.9	*213707_s_at*	*distal-less homeobox 5 (DLX5)*	*−31.8*
	215076_s_at	collagen, type III, alpha 1 (COL3A1)	38.9	*213721_at*	*SRY -box 2 (SOX2)*	*−27.3*
	206282_at	neurogenic differentiation 1 (NEUROD1)	29.9	*201215_at*	*plastin 3 (PLS3)*	*−25.6*
	206456_at	GABA receptor, alpha 5 (GABRA5)	26.4	*205501_at*	*phosphodiesterase 10A (PDE10A)*	*−25.5*
	204684_at	neuronal pentraxin I (NPTX1)	18.6	*205030_at*	*fatty acid binding protein 7, brain (FABP7)*	*−17.8*
	206596_s_at	neural retina leucine zipper (NRL)	17.9	*205330_at*	*meningioma 1 (MN1)*	*−15.6*
	202431_s_at	v-myc myelocytomatosis viral oncogene homolog (MYC)	12.2	*205350_at*	*cellular retinoic acid binding protein 1 (CRABP1)*	*−13.6*
	206047_at	guanine nucleotide binding protein 3 (GNB3)	12.0	*203889_at*	*secretogranin V (SCG5)*	*−13.1*
Group 4 *versus NFB*	206163_at	mab-21-like 1 (MAB21L1)	168.4	*221805_at*	*neurofilament, light polypeptide (NEFL)*	*−55.1*
	205445_at	prolactin (PRL)	21.0	*218692_at*	*syntabulin (SYBU)*	*−40.1*
	206373_at	Zic family member 1 (ZIC1)	14.9	*205330_at*	*meningioma 1 (MN1)*	*−35.6*
	202983_at	helicase-like transcription factor (HLTF)	11.3	*205691_at*	*synaptogyrin 3 (SYNGR3)*	*−28.3*
	221569_at	Abelson helper integration site 1 (AHI1)	7.1	*204081_at*	*neurogranin (NRGN)*	*−23.8*
	200934_at	DEK oncogene (DEK)	6.8	*212148_at*	*pre-B-cell leukemia homeobox 1 (PBX1)*	*−12.4*
	213029_at	nuclear factor I/B (NFIB)	6.6	*203548_s_at*	*lipoprotein lipase (LPL)*	*−11.8*
	218515_at	GC-rich sequence DNA-binding factor 1 (GCFC1)	6.1	*219415_at*	*tweety homolog 1 (TTYH1)*	*−11.6*
	218829_s_at	chromodomain helicase DNA binding protein 7 (CHD7)	5.9	*213924_at*	*guanine nucleotide binding protein (GNAL)*	*−11.1*
	209337_at	PC4 and SFRS1 interacting protein 1 (PSIP1)	5.2	*202668_at*	*ephrin-B2 (EFNB2)*	*−10.6*

fch – fold change, NFGM – neural fetal germinal matrix, NFB – neural fetal brain.

### Novel connections between granule neuron development and cell cycle regulation in Group 4 medulloblastoma

**Figure 4:**
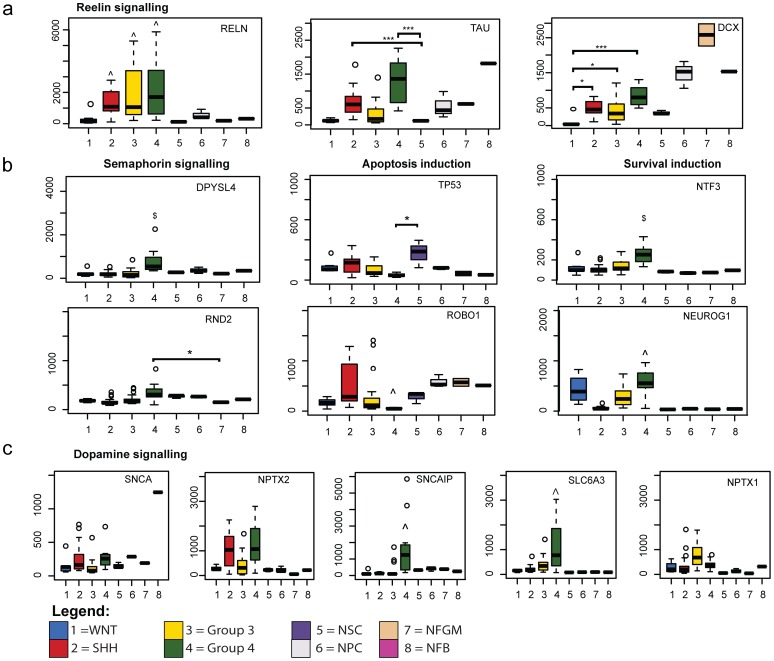
Granule neuron signatures of Group 4 medulloblastoma. a. Differential expression of reelin signaling members b. Semaphorin signaling pathway members and downstream mediators and c. Dopamine/α-synuclein signaling pathway members are shown for all sample groups using Box-Whisker plots. * −p<0.05, ** −p<0.01, *** −p<0.001, ∧ −p<0.05 compared to all control groups but not exclusive for medulloblastoma subgroup, NFB - normal fetal brain, NFGM - normal fetal germinal matrix, NPC - neural progenitor cells, NSC - neural stem cells.

The most prominent molecular feature of Group 4 medulloblastoma is the general up-regulation of neuronal lineage transcription factors [21]. Consistent with this, neuronal guidance and synaptic signaling pathways were strongly associated with Group 4 medulloblastoma in our analyses (Table. 1, Table S3). Several studies have suggested that the neuronal signatures characteristic of Group 4 medulloblastoma are reminiscent of active glutamatergic neurons (reviewed by Northcott et al. [31]), although this link has not been investigated in detail. With this in mind, we assessed key transiently activated neural transcription factors (*MATH1, NHLH1*), and other temporally activated genes (*NEUROD1, NHLH2, NFIB, LPPR4, DPYSL3, NEUROD2*) expressed throughout granule neuron differentiation, in the medulloblastoma subgroups (Fig. 3c). Alignment of the glutamatergic gene expression markers showed that SHH, Group 3, and Group 4 subgroups all express glutamatergic granule neuron genes, but from different temporal stages of development. SHH medulloblastoma resembled granule precursor cells (GPCs) in the external granule layer (EGL) around week 6 of human embryogenesis, whereas Group 3 resembled granule neuron precursors (GNPs) in the EGL around week 15, and Group 4 expressed genes that are only activated during late fetal development of the cerebellar EGL and cortex. We also observed activation of the Reelin pathway, another hallmark of granule neuron development in the cerebellum, in all medulloblastoma molecular subgroups, except for WNT tumors (Fig. 4a). Reelin pathway activation during the late embryonic to early fetal period of gestation has been observed in mouse model [32], and increasing expression of reelin pathway components, *TAU* and *DCX*, in neurodevelopmental tissues representing equivalent human stages was also observed in this study (Fig. 4a).

It is unclear how the neuronal developmental gene sets activated in Group 4 medulloblastoma are involved in the pathogenesis of this subgroup. When we investigated associated network clusters and exclusively differentially expressed transcripts in Group 4 medulloblastoma (Table 1, Table S2, S3), we found that several associated pathways contain co-expressed genes forming bridging pathway links. Semaphorin (co-expression network, p<1.23×10^−5^) and dopamine (intersect analysis, p<0.02) signaling pathways were exclusively enriched in Group 4 medulloblastoma and connected via co-expressed genes, *DPYSL4, RND2* and *SNCAIP* that are all part of the same network cluster (Fig. 4b,c). Both pathways were connected in the same co-expression cluster with *TAU* and *DCX* of the reelin pathway. Further, semaphorin pathway members, *RND2, DPYSL3*, *RAC1* and *FNBP1* are known to interact with apoptosis regulators *TP53* and *ROBO1*, as well as neuronal survival genes *NTF3* and *NEUROG1* during the late fetal synaptic pruning phase [33]–[36]. Whilst expression of apoptosis genes *TP53* and *ROBO1* was below the detection limit, the pro-survival genes *NTF3* and *NEUROG1* were over-expressed in Group 4 medulloblastoma, irrespective of control comparison (Fig. 4b). Considering that *NTF3* is also part of the same cluster family of the network, this implies a functional connection between deregulation of reelin, dopamine and cell cycle pathways that is worth exploring in the context of Group 4 pathogenesis.

## Discussion

**Figure 5:**
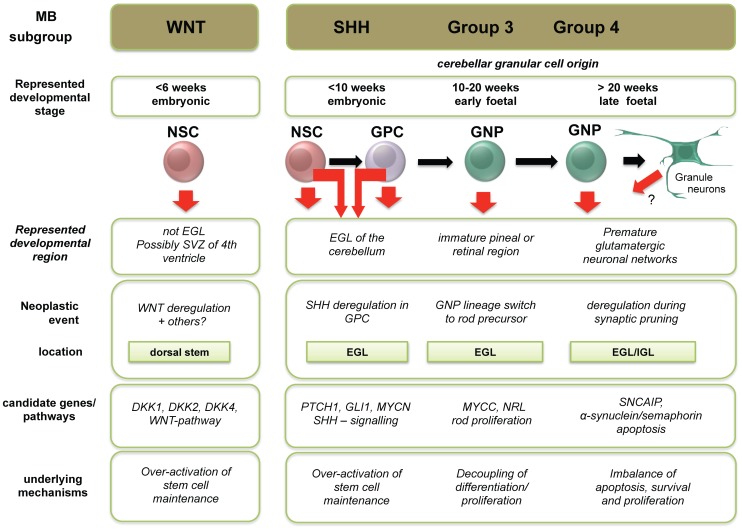
Schematic summary of the spatio-temporal alignment of medulloblastoma subgroups with proposed cells of origin. The summary shows novel concepts derived from this study integrated with existing medulloblastoma paradigms from the literature. *EGL – external granule layer, GPC  =  granule precursor cell, GNP – granule neuron precursor, IGL – internal granule layer, NSC – neural stem cell.*

The current evidence suggests that medulloblastoma subgroups arise in distinct regions of the cerebellum or cerebellar vicinity [6], [7] and are associated with characteristic genetic aberrations affecting the local cell of origin [5]. Our data revealed molecular relationships of medulloblastoma subgroups to distinct developmental control samples, and highlighted subgroup-specific deviation in gene expression from normal neuronal lineage differentiation programs that may contribute to medulloblastoma pathogenesis (Fig. 5). Extending on this idea, we hypothesised that shared expression features between medulloblastoma subgroups and specific controls may be indicative of the neuro-developmental stage and potentially, the cell of origin. WNT and SHH medulloblastoma consistently clustered closer to embryonic-derived cell types, whereas Group 3 and Group 4 medulloblastoma clustered closer to foetal samples. Consistent with the literature [3], WNT medulloblastoma expression signatures set them apart from the other three subgroups. Our data suggest that this may be linked to the relative inactivity of neuronal developmental pathways, reflected by reduced expression of glutamatergic granule neuron markers and reelin pathway members, which are strongly expressed in normal granule cell precursors during early cerebellar development and into post-natal stages [37]. We noted that several reelin pathway members are significantly over-expressed in SHH, Group 3 and Group 4 medulloblastoma, but are not expressed in the WNT subgroup. *RELN* mutations cause mis-alignment and degeneration of granule neurons in the cerebellar cortex [38], [39]. However, the oncogenic implications of reelin hyper-activation or ectopic expression during neuronal differentiation have not been determined and warrant further investigation.

In accord with previous reports [21], [22], [28], we identified distinct groups of co-expressed genes that were highly enriched for WNT signalling in WNT medulloblastoma, (including *DKK1, DKK2, DKK4, WIF1, LEF1* and *WNT16*). Similarly, over-expression of associated network clusters enriched for SHH pathway members, *GLI1, PTCH1* and *PTCH2*, was exclusive to human SHH medulloblastoma, and expression levels were significantly higher than in CD133^+^ NSCs, NPCs and all other controls used in this study. WNT and SHH signalling regulate NSC self-renewal and differentiation [40], and deregulation of these pathways is consistent with mouse models implicating a NSC cell of origin for at least some WNT and SHH subgroup medulloblastoma [14], [15].

Our data indicate that the expression profiles of SHH, Group 3 and Group 4 medulloblastoma are similar to cerebellar granule neuron gene expression signatures, but there are distinct differences in the specific pathways that are active. Molecular studies comparing the four medulloblastoma subgroups revealed that Group 3 medulloblastoma exclusively express photoreceptor genes normally associated with retinal development [8], [21], [28]. Our data significantly extend these findings, and indicate that the expression profiles of Group 3 medulloblastoma resemble that of rod precursor cells at week 15 of human retinal development. All Group 3 medulloblastoma expressed the transient rod precursor gene *NRL* at high levels, and most over-expressed *CRX* and *SAG*, with only a few expressing mature rod cell genes, *RCVRN* and *RHO*. The expression of the latter markers has been well documented in normal rod/cone specification during embryogenesis [41]. In contrast, the pre-rod-determination gene, *MITF*, and cone cell markers normally expressed after lineage commitment to cone cells [42], were not expressed in Group 3 medulloblastoma. *CRX* is a down stream target of the neuronal differentiation repressor *OTX2*. The latter is known to be over-expressed in medulloblastoma [43] raising the possibility that over-expression of *OTX2* causes a premature halt in neuronal differentiation. While *OTX2* and *CRX* are both expressed in the retina, pineal region and EGL from E16 of murine development (equiv. week 10 in human), *CRX* expression regresses to the pineal gland from E18 (equiv. to week 15 human) and is not detectable in normal mouse cerebellum at all after this time [44]. Therefore, it seems possible that during this developmental window, *OTX2* and *CRX* expression deviates from normal differentiation patterns generating phenotypes more reminiscent of a retinal or pineal cell. The fact that *CRX* is expressed by Group 3 medulloblastoma as well as most pineal tumours [45], and that Group 3 medulloblastoma and pineoblastoma are often histologically indistinguishable [45], [46] and share a poor prognosis despite intensive therapy, supports the idea of a molecular relationship between the two. For medulloblastoma, the relationship between rod receptor signatures and neoplastic transformation remains unclear, but we speculate that deregulated differentiation and lineage switch to rod fate may play an important role.

Our data indicate that the expression profiles of Group 4 medulloblastoma are similar to cerebellar glutamatergic granule neurons at late foetal developmental stage (> week 20). Group 4 medulloblastoma expressed late granule neuron markers and reelin pathway members most highly, which are normally expressed in granule cell precursors during cerebellar development and into post-natal stages [37]. Most of our data demonstrating dominant expression of genes regulating neuronal migration, differentiation and synaptic development is consistent with previous findings [21]. We confirmed *SNCAIP* as exclusively expressed in Group 4 medulloblastoma [47], and we also identified *SLC6A3* as a significantly over-expressed and highly connected pathway member. In glutamatergic neurons, *SNCAIP* and *SLC6A3* are involved in the regulation of dopamine concentration [48]. Over-expression of *SNCA* or *SNCAIP* in GPCs has been shown to promote GPC proliferation and protection from apoptosis [49]–[52]. Interestingly, a recent report describing epigenetic repression of the dopamine receptor family member, DRD4, in primary medulloblastoma adds further support for a significant role for deregulated dopamine signalling in medulloblastoma pathogenesis [53]. Additional novel findings from our analysis indicate that Group 4 medulloblastoma over-express semaphorin signalling molecules, including *DPYSL3* and *DPYSL4*, which regulate neuronal differentiation and apoptosis in the context of synaptic pruning [54]. Overall, our results build significantly on previous data and implicate an important connection between dopamine, semaphorin signalling and cell cycle deregulation in the pathogenesis of Group 4 medulloblastoma. These pathways may constitute promising therapeutic targets to combat medulloblastoma angiogenesis, growth and metastases [53], [55], [56].

The consensus in the field is that the four medulloblastoma subgroups will be further subdivided as more genomic data become available, and more refined animal models are developed. Additional subtypes will likely be derived from distinct intra or extra cerebellar cells of origin. Clearly, this hetereogeneity complicates comparative analysis of medulloblastoma expression profiles to those of selected “normal” cells, including whole human foetal cerebellum. In this study we assessed highly purified cell populations from cultured neurospheres, as well as heterogeneous cell populations from resected tissues. Although the transcriptional profiles of cell lines maintained *in vitro* will inevitably differ to some extent from intra or extra cerebellar NSCs within the normal developing brain [57], the human ESC-derived NSCs analysed in our study self renew and differentiate normally *in vitro*, and when transplanted *in vivo* [58]. Neurospheres are cultured in EGF and FGF, which is known to drive cell proliferation via SHH, WNT, and NOTCH signalling [59]. However, in this study, activation of these pathways correlated most closely with developmental stage rather than culture *versus* non-culture status. Overall, these data suggest that ESC derived NSC/NPCs represent a reasonable and practical option for comparison to medulloblastoma transcriptional profiles and characteristics.

Our data provide a valuable framework for the design of more refined cell of origin studies that are currently lacking in the human context, particularly for Group 3 and Group 4 medulloblastoma (Fig. 5). For example, our data support the analysis of cell populations derived from GPCs, as well as retinal and pineal neuronal precursors, to address potential aberrant trans-lineage differentiation in the pathogenesis of Group 3 medulloblastoma. Indeed, GPCs and GNPs, as well as GNPs and rod precursor cells generated from ESC-derived NSCs [60]–[62] represent promising candidate populations for further genetic comparisons. In addition, as described by our group previously [26], human ESC-derived NSCs may in turn be a useful *in vitro* system to model medulloblastoma pathogenesis *in vitro* and potentially *in vivo* using orthotopic xenografts. Functional testing of proposed cancer driver genes in putative human cell of origin populations will be a crucial step towards the identification of novel therapeutic targets in the various medulloblastoma molecular subgroups.

## Supporting Information

Figure S1 **Variation distribution and length of principle component vectors for combined medulloblastoma cohorts and all developmental controls.** Individual and accumulative total variation for the largest principle components as indicated. Discriminative components are marked in red.
(DOCX)

Figure S2 **Co-expression modules and developmental alignment.** Medulloblastoma cluster families of the co-expression network were generated using Eigengenes. Cluster family E that associated closest with alignment orders was emphasized in red shading. *NFB – normal foetal brain, NFGM – normal foetal germinal matrix, NPC – neural progenitor cells, NSC – neural stem cells*.
(DOCX)

Figure S3 **Expression of NOTCH pathway members in medulloblastoma subgroups and control.** Most NOTCH signalling members were significantly lower expressed in medulloblastoma subgroups than in NSC-rich samples. Expression levels were graphed using Box-Whisker plots. ¢ −*p<0.05 compared to all medulloblastoma subgroups. NFB – normal foetal brain, NFGM – normal foetal germinal matrix, NPC – neural progenitor cells, NSC – neural stem cells*.
(DOCX)

Figure S4 **Photoreceptor lineage expression alignment.** Expression levels of transcripts known to be actively transcribed during cone lineage specification was presented as mean and standard deviation for all sample groups. *NFB – normal foetal brain, NFGM – normal foetal germinal matrix, NPC – neural progenitor cells, NSC – neural stem cells*.
(DOCX)

Table S1 **Details of clinical cohort and developmental samples.**
(DOCX)

Table S2 **Transcripts over-expressed in all medulloblastoma subgroups irrespective of control group comparison.**
(DOCX)

Table S3 **Exclusive transcripts associated with pathway enrichment in medulloblastoma subgroups.**
(DOCX)
