# Supplementary figures and images for: Gene Expression Analyses of the Spatio-Temporal Relationships of Human Medulloblastoma Subgroups during Early Human Neurogenesis
Source: PLoS One. 2014 Nov 20;9(11):e112909. doi: 10.1371/journal.pone.0112909 (PMC4239019; doi:10.1371/journal.pone.0112909)

Figure S1


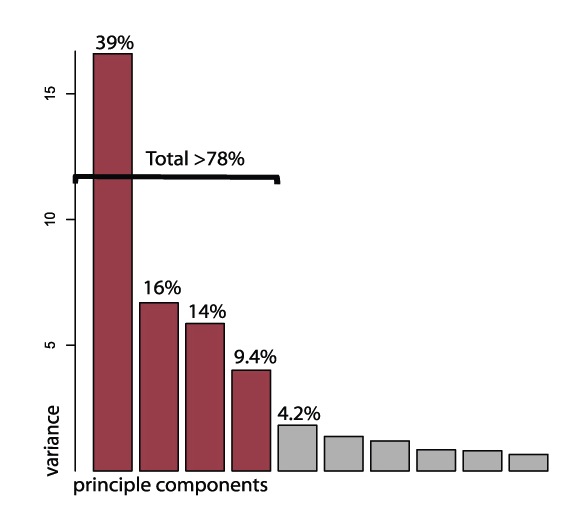

Supplement: Figure S1 — Variation distribution and length of principle component vectors for combined medulloblastoma cohorts and all developmental controls. Individual and accumulative total variation for the largest principle components as indicated. Discriminative components are marked in red. (DOCX) [file pone.0112909.s001.docx]

Figure S2


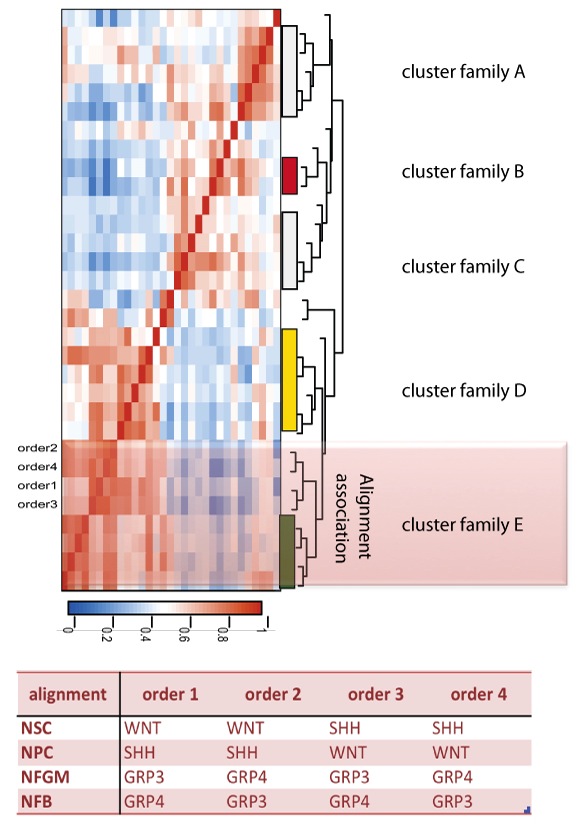

Supplement: Figure S2 — Co-expression modules and developmental alignment. Medulloblastoma cluster families of the co-expression network were generated using Eigengenes. Cluster family E that associated closest with alignment orders was emphasized in red shading. NFB – normal foetal brain, NFGM – normal foetal germinal matrix, NPC – neural progenitor cells, NSC – neural stem cells. (DOCX) [file pone.0112909.s002.docx]

Figure S4


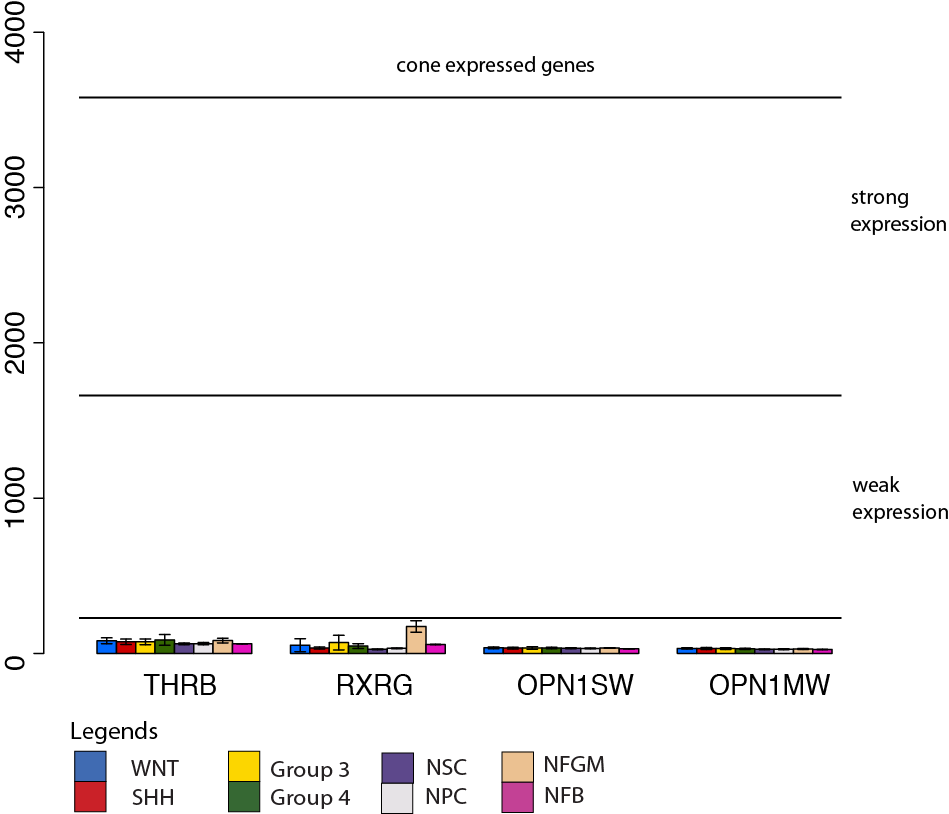

Supplement: Figure S4 — Photoreceptor lineage expression alignment. Expression levels of transcripts known to be actively transcribed during cone lineage specification was presented as mean and standard deviation for all sample groups. NFB – normal foetal brain, NFGM – normal foetal germinal matrix, NPC – neural progenitor cells, NSC – neural stem cells. (DOCX) [file pone.0112909.s004.docx]
