# Supplementary material for: Gene Expression Analyses of the Spatio-Temporal Relationships of Human Medulloblastoma Subgroups during Early Human Neurogenesis
Source: PLoS One. 2014 Nov 20;9(11):e112909. doi: 10.1371/journal.pone.0112909 (PMC4239019; doi:10.1371/journal.pone.0112909)
Supplement: Table S2 — Transcripts over-expressed in all medulloblastoma subgroups irrespective of control group comparison. (DOCX) [file pone.0112909.s006.docx]

**Table S2**

**

*fch – fold change, NFB - normal foetal brain, NFGM - normal foetal germinal matrix, NPC - neural precursor cells, NSC - neural stem cells*
